# Doxapram hastens the recovery following total intravenous anesthesia with dexmedetomidine, propofol and remifentanil

**DOI:** 10.3892/etm.2015.2249

**Published:** 2015-02-02

**Authors:** HUAN-LIANG WANG, SHU-HAI TANG, XUE-QIN WANG, WEN-HUA GONG, XIAO-MEI LIU, WEI-FU LEI

**Affiliations:** Department of Anesthesiology, Qilu Hospital, Shandong University, Jinan, Shandong 250012, P.R. China

**Keywords:** doxapram, dexmedetomidine, propofol, remifentanil, obstructive sleep apnea

## Abstract

Dexmedetomidine is a suitable sedative for awake fiberoptic intubation in patients with obstructive sleep apnea (OSA). However, previous studies have shown that dexmedetomidine delays recovery from propofol-remifentanil anesthesia. This study aimed to determine whether doxapram may hasten the recovery following dexmedotomidine-propofol-remifentanil anesthesia. Sixty patients scheduled for uvulopalatopharyngoplasty with total intravenous anesthesia were randomized to two groups according to the medicine given at the end of surgery. These were the doxapram (1 mg/kg) and control (normal saline) groups (n=30 per group). The primary outcome was the time to eye opening on verbal command. The time to return to spontaneous breathing, to hand squeezing in response to verbal command, to extubation of the trachea, and the heart rate (HR), bispectral index (BIS) values, respiratory rate (RR) and pulse oximetry values were also recorded and compared. The time to return to spontaneous breathing (5.2±2.9 vs. 11.7±3.4 min, P<0.001), eye opening (9.3±4.7 vs. 15.9±6.3 min, P<0.001), hand squeeze to command (11.8±6.5 vs. 17.6±7.7 min, P=0.0026) and extubation (14.2±7.8 vs. 19.2±9.6 min, P=0.0308) were significantly shorter in the doxapram group compared with the control group. BIS scores (at 3–14 min), RR (at 4–10 min) and HR (at 2–13 min) were significantly higher in the doxapram group compared with those in the control group (P<0.05). Doxapram hastens the recovery from dexmedetomidine-propofol-remifentanil anesthesia in patients undergoing uvulopalatopharyngoplasty, and may benefit patients with OSA.

## Introduction

General anesthesia with a secured airway is recommended for patients undergoing uvulopalatopharyngoplasty for obstructive sleep apnea (OSA) (1). Patients with OSA are at increased perioperative risk due to their susceptibility to the respiratory depressant and airway effects of sedatives, opioids and inhaled anesthetics, which can be attributed to their propensity for airway collapse and sleep deprivation. Therefore, securing the airway and reducing the postoperative respiratory compromise should be considered when selecting anesthetics.

Dexmedetomidine, a highly selective α2-adrenergic receptor agonist, provides arousable sedation similar to that of natural sleep and preserves spontaneous respiration even at large doses, making it suitable for sedation in awake intubation procedures such as fiberoptic intubation (FOI) (2–4). In addition, dexmedetomidine may be a useful adjuvant during general anesthesia, particularly for OSA patients, by promoting hemodynamic stability and decreasing the doses of anesthetics and analgesics, which may therefore allow early recovery and reduce potential postoperative respiratory compromise (5,6). However, studies have shown that dexmedetomidine delays recovery from propofol or propofol-remifentanil anesthesia (7,8). Propofol and propofol-remifentanil are commonly used anesthetics for patients with OSA undergoing uvulopalatopharyngoplasty (9,10).

Doxapram is a respiratory and central nervous system (CNS) stimulant, which has comprehensive effects on peripheral and central chemoreceptors and could potentially hasten the recovery from several types of anesthetics (11–19). Therefore, it was hypothesized that doxapram may accelerate the recovery following dexmedotomidine-propofol-remifentanil anesthesia. In this study, the aim was to determine whether doxapram hastens the recovery in OSA patients following dexmedetomidine-propofol-remifentanil anesthesia.

## Patients and methods

### Patient eligibility

This double-blind, randomized prospective study was approved by the Ethics Committee of Qilu Hospital of Shandong University (Jinan, China) and registered as a clinical trial (http://www.chictr.org/; identifier, ChiCTR-TRC-13003346). Written, informed consent was obtained from 60 adult patients with American Society of Anesthesiologists (ASA) class I and II physical status scheduled for elective uvulopalatopharyngoplasty for OSA. Polysomnograms were performed in all patients (Alice 4™; Respironics Inc., Pittsburgh, PA, USA) and the records were staged manually according to standard criteria by the same skilled technician. Respiratory events were scored according to the American Academic Sleep Medicine (AASM) criteria: Apnea was defined as complete cessation of airflow lasting for ≥10 sec; hypopnea was defined as either a ≥50% reduction in airflow for ≥10 sec, or a <50% but discernible reduction in airflow accompanied either by a reduction in oxyhemoglobin saturation of ≥4% or an arousal. The apnea-hypopnea index (AHI) was defined as the number of events of apnea and hypopnea per hour during sleep time, based on the results of the overnight polysomnographs (PSGs). If the AHI was ≥5/h, the patient was diagnosed as positive for OSA. The patients were excluded if they had bradycardia [<50 beats per min (bpm)], hypotension (systolic blood pressure <90 mmHg), hepatic impairment, had taken a α2-adrenoceptor agonist or antagonist within the previous 14 days, were contraindicated for nasal intubation, intolerant or allergic to the study drug, or refused to be involved in the study.

### Patient grouping

Patients were randomized into two groups according to a computer-generated table of random numbers. The doxapram group (n=30) received doxapram (Jiangsu Nhwa Pharmaceutical Corporation Ltd., Xuzhou, China) 1 mg/kg intravenously (i.v.), and the control group (n=30) received isovolumic normal saline i.v. Study drugs were prepared by an anesthesia nurse. The anesthesiologist and the subjects were unaware of group identities.

### Surgical anesthesia

Atropine (0.5 mg; Minsheng Pharmaceutical Group Co., Ltd., Hangzhou, China) was administered intramuscularly as premedication 30 min prior to the patient’s arrival in the operating room. On arrival in the operating room, routine monitors were applied to each subject, including continuous electrocardiogram, peripheral pulse oximeter, non-invasive blood pressure monitor, end-tidal CO_2_ monitor and bispectral index (BIS) monitor (S/5; GE Healthcare Finland Oy, Helsinki, Finland). Following the recording of baseline vital signs and BIS values, dexmedetomidine (Jiangsu Hengrui Medicine Co. Ltd., Lianyungang, China) was administered as a loading dose of 1.0 μg/kg over 10 min followed by a continuous infusion of 0.7 μg/kg/h until a Ramsey score of 3 was achieved. Topical lidocaine (Shanghai Zhaohui Pharmaceutical Co., Ltd., Shanghai, China) was administered for an uneventful FOI.

Following the identification of exhaled CO_2_ by infrared spectroscopy, general anesthesia was induced with 2 mg/kg propofol (AstraZeneca, London, UK) i.v., and initial muscle relaxation was achieved with 50 mg atracurium (Jiangsu Hengrui Medicine Co., Ltd.) i.v. Ventilation was adjusted to maintain end-tidal CO_2_ values between 35 and 40 mmHg using an inspiratoy O_2_ fraction of0.5.

Anesthesia was titrated with propofol to maintain the BIS scores in the range of 50±10, with remifentanil infusion (0.1–0.25 μg/kg/min; Yichang Renfu Pharmaceutical Co., Ltd., Yichang, China) to maintain the heart rate (HR) between 50 and 80 bpm and atracurium (5–10 μg/kg/min) to maintain a train-of-four (TOF) stimulation value of 0 [TOF-Watch^®^ SX; Organon (Ireland) Ltd., Dublin, Ireland]. Neostigmine (0.05 mg/kg; Shandong Tianfu Pharmaceutical Factory, Zibo, China) and atropine 0.01 mg/kg i.v.) were administered 10 min prior to the end of surgery to allow for the return to spontaneous breathing. Propofol and remifentanil were discontinued at the end of surgery and then the study drug (doxopram or saline) was administered i.v. over 1 min.

### Recovery procedure

The name of the patient was called every 30 sec, and the patient was asked ‘Are you awake? Open your eyes.’ The time from the end of the general anesthesia to eye opening was measured. Tracheal extubation was performed when the patients achieved a regular breathing pattern and were able to follow the verbal command to squeeze the anesthesiologist’s hand.

The following parameters were evaluated by an anesthesiologist who was unaware of study group allocations: Time to return to spontaneous breathing, eye opening on verbal command, hand squeezing in response to verbal command, and time to extubation of the trachea from the end of general anesthesia. HR, systolic blood pressure, BIS values and SpO_2_ values were determined prior to surgery, at 5-min intervals during surgery, and then at each minute after the injection of the study drugs for 16 min. The respiratory rate (RR) was also recorded from the time of study drug injection to the time of extubation. Modified Aldrete scores (20) were evaluated every 5 min until a score of 9 was reached, and then the patient was discharged from the operating room.

**Figure 1:**
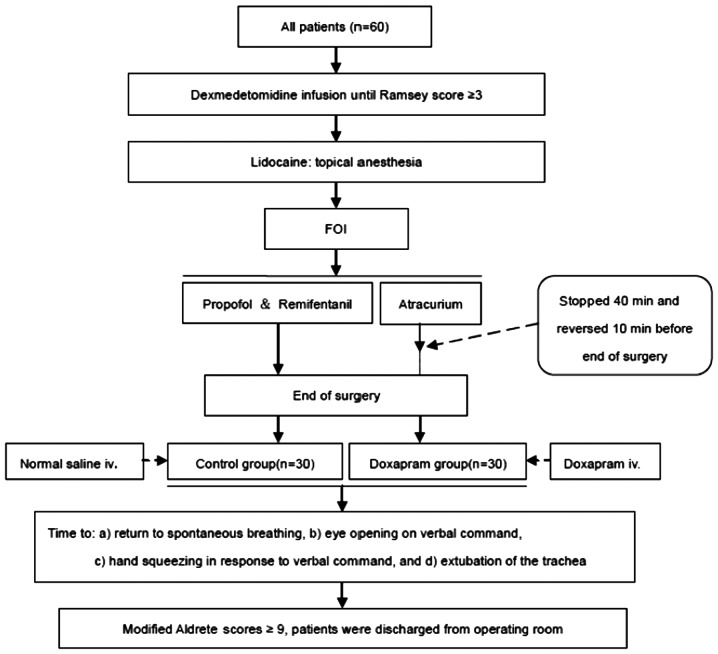
Flow chart of the anesthesia process. FOI, fiberoptic intubation.

The anesthesia process and the treatment of patients are schematically illustrated in Fig. 1.

Recall, awareness during anesthesia or abnormal psychological feeling during emergence was recorded at 24 h after surgery. The incidences of reintubation, hypoxemia, myocardial infarction, arrhythmia, delirium, thromboembolism were also recorded at 24 h after surgery.

### Statistical analysis

The time to achieve eye opening on verbal command was defined as the primary end-point of this study. The time to return to eye opening in the control group was 15.0±6.2 min in a pilot study of 10 patients, and it was assumed that the standard deviation (SD) in the test group was equal to that of the control group. A difference of 5 min to eye opening was set between groups. At least 25 patients per group were required to provide 80% power to detect this difference at α=0.05. Assuming the possibility of patients being excluded from the study, 30 patients were enrolled per group. For continuous variables, the distribution of the data was first evaluated using the Kolmogorov-Smirnov test for normality. The normally distributed data are presented as the mean ± SD, and significance was tested via the Student’s t-test. The non-normally distributed data were analyzed via the Mann-Whitney U test. Descriptive variables were subjected to Chi-square analysis. In all tests, P<0.05 was considered to indicate a statistically significant difference.

## Results

### Patient and treatment variables

There were no significant differences in demographic characteristics, duration of anesthesia and doses between the doxapram and control groups (Table I).

### Recovery parameters

The time to return to spontaneous breathing, eye opening, hand squeeze on command, and extubation were observed to be significantly shorter in the doxapram group (P<0.05) compared with those in the control group (Table II).

**Figure 2:**
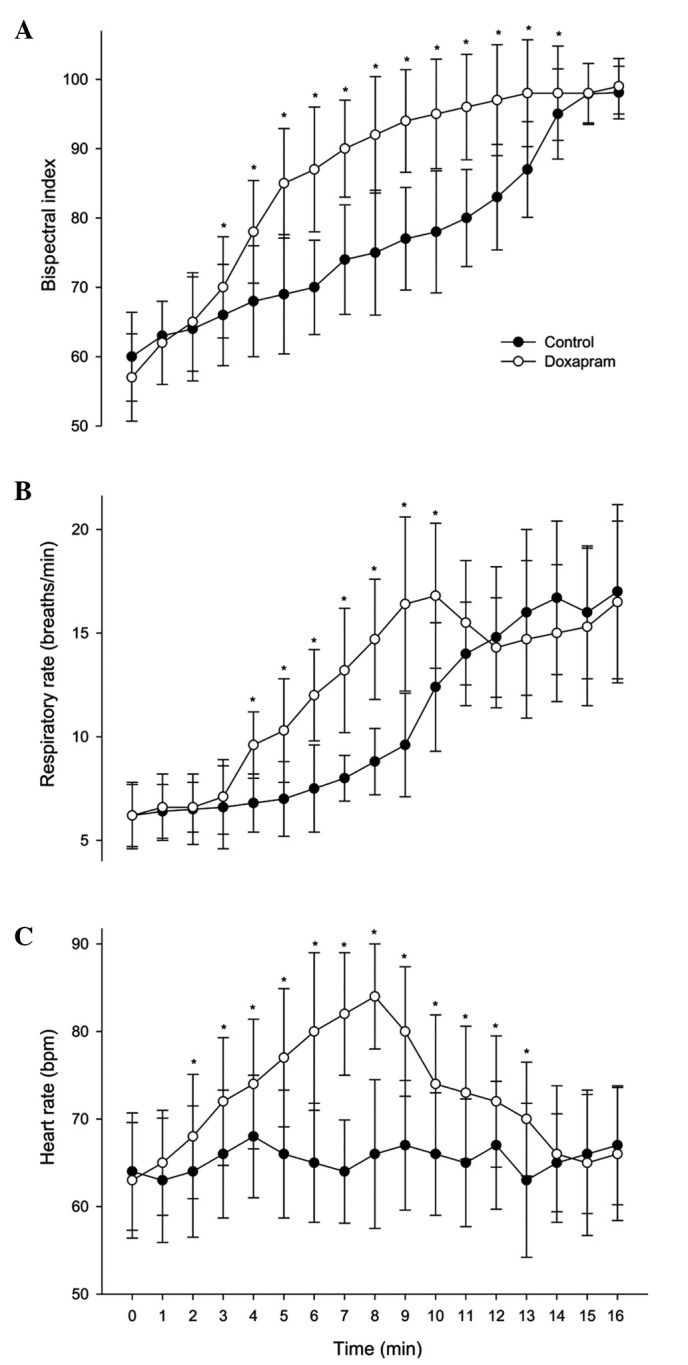
Changes in (A) bispectral index, (B) respiratory rate and (C) heart rate following the administration of doxapram. ^*^P<0.05 vs. control. bpm, beats per minute.

Following treatment, the BIS scores were significantly higher in the doxapram group at 3–14 min compared with those in the control group (P<0.05; Fig. 2A). The doxapram group had significantly higher RR and HR values compared with those in the control group at 4–10 min (in RR) and at 2–13 min (in HR) (P<0.05; Fig. 2B and C). No significant differences were identified in BIS values, end-tidal CO_2_, HR and systolic blood pressure during the anesthesia and post-anesthesia periods between the two groups.

Three patients in the control group and two in the doxapram group experienced a HR <50 bpm, and all these patients achieved a HR of 50 bpm within 5 min without treatment during dexmedetomidine infusion. All patients who received doxapram recovered from anesthesia smoothly without unpleasant psychological effects, remained calm and could respond well to commands. There were no differences in the modified Aldrete score between the two groups.

Hypoxemia (SpO_2_ <90%) occurred in four patients in the control group and in five in the doxapram group but only one patient from the control group required reintubation. No other adverse effects were observed at 24 h after surgery.

## Discussion

The main finding of this study is that the recovery from dexmedetomidine-propofol-remifentanil anesthesia in patients with OSA undergoing elective uvulopalatopharyngoplasty is hastened by doxapram (1 mg/kg i.v.). Rapid and complete recovery from general anesthesia benefits patients with OSA following uvulopalatopharyngoplasty (6). However, dexmedetomidine has been demonstrated to delay the recovery from propofol anesthesia when used to maintain the anesthesia or to induce anesthesia in minor surgery due to its longer half-life (≥2 h) (7–9). Doxapram has been used to treat drug-induced post-anesthetic CNS depression, including that arising from the use of opioids and propofol (17,18). A 1-mg/kg dose of doxapram is commonly recommended for intravenous injection (18,19). The present study indicated that doxapram is effective in reversing the anesthetic effects of dexmedetomidine-propofol-remifentanil.

The present study also demonstrated that doxapram administration caused a rapid recovery of BIS, similar to that in the reversal of sevoflurane and propofol-remifentanil anesthesia (18,19). Wang *et al* demonstrated that a loading dose of dexmedetomidine of 1.0 μg/kg over 10 min followed by infusion at 0.5 μg/kg/h decreased the BIS values under stepwise propofol target-controlled infusion (21). Previous studies have also indicated that BIS correlates well with the hypnotic and sedative effects of various anesthetic agents, including isoflurane, sevoflurane, midazolam and propofol (22,23). The data from the present study demonstrated that BIS correlated well with the levels of consciousness under the circumstance of co-administration of dexmedetomidine with propofol-remifentanil. The recovery effect of doxapram may be associated with rapid recovery of BIS values resulting from its nonspecific and extensive CNS stimulant properties.

Some adverse effects of doxapram have been reported, including tachycardia, cardiac arrhythmia, hypertension, anxiety reactions, hallucinations, excitation, panic attacks and even cerebrovascular accident (14,16,17,24). In the present study, 1 mg/kg doxapram was used, a dose that has previously been demonstrated to be effective in reversing the depressant effects of anesthetic without adverse responses (15–19). All patients who received doxapram remained calm following extubation of the trachea, in a similar manner to those in the control group. It appears that the sedative effect of dexmedetomidine continues beyond the time of extubation. The complications associated with anesthesia in patients with OSA undergoing uvulopalatopharyngoplasty were not different between groups in the present study.

There are several limitations in this study. Firstly, a dexmedotomidine-propofol-remifentanil group was not provided as a negative control in this study to demonstrate the more prolonged recovery time associated with dexmedetomidine co-administration. Secondly, serum dexmedetomidine, propofol or remifentanil concentrations were not measured, precluding the ability to distinguish pharmacokinetic interactions from pharmacodynamic interactions. Thirdly, the data could not distinguish which part of the co-administration of dexmedetomidine, propofol and remifentanil was the main target of doxapram action. The inclusion of a control group without dexmedetomidine will be used in future studies to clarify the mechanism by which doxapram accelerates the recovery of patients with OSA following total intravenous anesthesia (TIVA).

In conclusion, a single dose administration of doxapram (1 mg/kg i.v.) at the end of TIVA hastens the early recovery from dexmedetomidine-propofol-remifentanil anesthesia in OSA patients undergoing uvulopalatopharyngoplasty without appreciable side-effects. Considering the benefits resulting from rapid and clear-headed emergence in OSA patients, this study provides helpful guidance on the clinical management of patients with OSA undergoing uvulopalatopharyngoplasty.

## Figures and Tables

**Table I:** Demographic and clinical data.

Variable	Control	Doxapram
Age (years)	38.6±9.7	37.6±9.8
Body height (cm)	179.5±16.7	179.2±17.0
Body weight (kg)	86.9±17.6	87.6±16.9
Anesthesia duration^a^ (min)	90.8±20.8	91.5±21.7
Dexmedetomidine (μg)	136.6±31.8	135.3±32.6
Propofol (mg)	577.8±130.6	584.8±142.0
Remifentanil (μg)	646.5±110.2	672.4±98.4
Atracurium (mg)	96.7±20.4	97.5±25.7

a Time from the injection of anesthesia to the withdrawal of anesthesia. Data are expressed mean ± standard deviation (n=30 per group).

**Table II:** Recovery parameters.

	Time for recovery (min)			
			
Recovery parameter	Control	Doxopram	t-value	P-value
Spontaneous breathing	11.7±3.4	5.2±2.9^a^	7.9668	<0.001
Eye opening	15.9±6.3	9.3±4.7^a^	4.5992	<0.001
Response to command	17.6±7.7	11.8±6.5^a^	3.1526	0.0026
Extubation of trachea	19.2±9.6	14.2±7.8^a^	2.2140	0.0308

a P<0.05 compared with the control group. Data are expressed as mean ± standard deviation (n=30 per group).
